# Trial Analysis of Brain Activity Information for the Presymptomatic Disease Detection of Rheumatoid Arthritis

**DOI:** 10.3390/bioengineering11060523

**Published:** 2024-05-21

**Authors:** Keisuke Maeda, Takahiro Ogawa, Tasuku Kayama, Takuya Sasaki, Kazuki Tainaka, Masaaki Murakami, Miki Haseyama

**Affiliations:** 1 Data-Driven Interdisciplinary Research Emergence Department, Hokkaido University, N-13, W-10, Kita-ku, Sapporo 060-0813, Japan; maeda@lmd.ist.hokudai.ac.jp; 2 Faculty of Information Science and Technology, Hokkaido University, N-14, W-9, Kita-ku, Sapporo 060-0814, Japan; ogawa@lmd.ist.hokudai.ac.jp; 3 Department of Pharmacology, Graduate School of Pharmaceutical Sciences, Tohoku University, 6-3 Aramaki-Aoba, Aoba-ku, Sendai 980-8578, Japan; k.task.k.125@gmail.com (T.K.); t.sasaki.0224@gmail.com (T.S.); 4 Department of Neuropharmacology, Tohoku University School of Medicine, 4-1 Seiryo-machi, Aoba-ku, Sendai 980-8575, Japan; 5 Department of System Pathology for Neurological Disorders, Brain Research Institute, Niigata University, 1-757 Asahimachi-dori, Chuo-ku, Niigata 951-8585, Japan; kztainaka@bri.niigata-u.ac.jp; 6 Division of Molecular Psychoimmunology, Institute for Genetic Medicine and Graduate School of Medicine, Hokkaido University, Kita-15, Nishi-7, Kita-ku, Sapporo 060-0815, Japan; murakami@igm.hokudai.ac.jp; 7 Division of Molecular Neuroimmunology, National Institute for Physiological Sciences, Myodaiji, Okazaki 444-8585, Japan; 8 Group of Quantum Immunology, National Institute for Quantum and Radiological Science and Technology (QST), 4-9-1 Anagawa, Inage 263-8555, Japan; 9 Institute for Vaccine Research and Development (HU-IVReD), Hokkaido University, Kita-21, Nishi-11, Kita-ku, Sapporo 001-0021, Japan

**Keywords:** presymptomatic disease, rheumatoid arthritis, brain activity, missing data, canonical correlation analysis

## Abstract

This study presents a trial analysis that uses brain activity information obtained from mice to detect rheumatoid arthritis (RA) in its presymptomatic stages. Specifically, we confirmed that F759 mice, serving as a mouse model of RA that is dependent on the inflammatory cytokine IL-6, and healthy wild-type mice can be classified on the basis of brain activity information. We clarified which brain regions are useful for the presymptomatic detection of RA. We introduced a matrix completion-based approach to handle missing brain activity information to perform the aforementioned analysis. In addition, we implemented a canonical correlation-based method capable of analyzing the relationship between various types of brain activity information. This method allowed us to accurately classify F759 and wild-type mice, thereby identifying essential features, including crucial brain regions, for the presymptomatic detection of RA. Our experiment obtained brain activity information from 15 F759 and 10 wild-type mice and analyzed the acquired data. By employing four types of classifiers, our experimental results show that the thalamus and periaqueductal gray are effective for the classification task. Furthermore, we confirmed that classification performance was maximized when seven brain regions were used, excluding the electromyogram and nucleus accumbens.

## 1. Introduction

According to the World Health Organization, the average life expectancy has increased by 5–10 years over the last two decades (from 2000 to 2019). Despite the concurrent increase in healthy life expectancy, the gap between overall and healthy life expectancy remains at approximately 10 years [1]. Longer healthy life expectancy is expected to improve quality of life, contribute to the overall revitalization of society, and reduce medical costs. In order to increase healthy life expectancy, it is essential to implement preventive measures at the “presymptomatic” stage, addressing health concerns before the onset of disease [2,3,4,5].

In recent years, machine learning has been significantly used in presymptomatic disease research, particularly for detecting early signs of Alzheimer’s disease, which increases in risk with age [2,4]. Despite these advancements, no established technology exists for pre-emptively detecting arthropathies such as rheumatoid arthritis (RA). Our study pioneers the development of a technology capable of identifying individuals at risk of developing RA. Previous studies on Alzheimer’s disease have demonstrated the efficacy of brain activity information, including electromyogram (EEG), functional near-infrared spectroscopy, and local field potential (LFP) in presymptomatic disease detection [6]. Therefore, it is expected that leveraging brain activity information will enable the accurate identification of presymptomatic individuals with RA.

Cytokines cause RA [7]. In order to verify the possibility of detecting individuals with RA using brain activity information, it is essential to develop the ability to classify F759 mice, which depend on the inflammatory cytokine IL-6, and healthy wild-type mice. Furthermore, determining the specific brain regions involved in RA is crucial. Although the direct analysis of brain activity information is necessary, specific brain regions may have missing information because of physical constraints, such as electrode insertion-related issues [8,9,10]. Consequently, extracting information about RA induction from incomplete brain activity data is a complex task. The primary objective of this study is to fill the gaps in brain activity information, enabling the analysis of the relationship between the data obtained from various brain regions, classification performance, and the identification of brain regions crucial for RA detection.

In this study, we conducted a trial analysis for the early detection of presymptomatic RA using missing value completion techniques for brain activity information and correlation analysis capable of assessing multiple aspects of brain activity. Specifically, we used a matrix factorization-based approach to address missing brain activity information, confirming its efficacy in complementing missing biological data, including behavior [11] and brain [12] data. In order to identify crucial brain regions for classifying F759 and wild-type mice, we employed canonical correlation analysis, a method widely used for analyzing various brain activity information [13,14,15,16,17]. Specifically, we used supervised multiview canonical correlation analysis (sMVCCA) [18], which is capable of handling multiple types of information. Subsequently, we calculated cross-loadings using features obtained from sMVCCA because this calculation is a powerful tool for revealing the potential relationships between biological information and other factors [19,20,21]. Thus, we identified important brain regions contributing to the classification of F759 and wild-type mice. Furthermore, by constructing a machine learning-based classification model using brain activity information, we clarified the relationship between information derived from different brain regions and classification performance.

## 2. Data Acquisition of Brain Activities and Feature Extraction

This section outlines the process of acquiring data from mice, as detailed in Section 2.1, and subsequently discusses the procedure for calculating features from the obtained data in Section 2.2.

### 2.1. Brain Activity Data Acquisition

#### 2.1.1. Animals

All mice used in this study were 8–16 weeks old and had pre-operative weights of 25–35 g. Male C57BL/6 J wild-type mice were purchased from SLC, Inc. (Shizuoka, Japan). The F759 mouse line carrying human gp130 (S710L) has been previously established [22]. In brief, the F759 mice underwent genetic modification involving the replacement of the intracellular region of the mouse gp130 gene, a gene responsible for encoding a signal transducer within the IL-6 receptor complex, with an intracellular mutant human gp130 cDNA (Y759F). This mutation inhibits SOCS3-mediated negative feedback, resulting in enhanced activation of STAT3 after IL-6 stimulation. Consequently, the injection of IL-6 and IL-17 into the ankle joints of F759 mice activates the IL-6 amplifier, improving the activation of the NFkB pathway in nonimmune cells, including synovial cells, ultimately resulting in the development of rheumatoid-like arthritis [23]. The mice were housed in a vivarium at a controlled temperature (22 ± 1 °C) and humidity (55 ± 5%), with a 12:12 h light/dark cycle (lights on from 8 am to 8 pm). The mice were provided ad libitum access to food and water and were individually housed.

#### 2.1.2. Surgery

The standard surgical procedures were similar to those described in previous studies [24,25,26]. The mice were anesthetized with 1–2% isoflurane gas in air and then fixed in a stereotaxic instrument equipped with two ear bars and a nose clamp. Craniotomy was performed on the left hemisphere at specific co-ordinates: anterior cingulate cortex (ACC; 1.1 mm anterior and 0.2 mm lateral to the bregma), prelimbic cortex (PL; 1.7 mm anterior and 0.2 mm lateral to the bregma), nucleus accumbens (NAc; 0.8 mm anterior and 0.8 mm lateral to the bregma), amygdala (AMY; 0.8 mm posterior and 3.0 mm lateral to the bregma), primary somatosensory cortex (S1; 1.4 mm posterior and 2.1 mm lateral to the bregma), thalamus (THL; 2.1 mm posterior and 1.3 mm lateral to the bregma), and periaqueductal gray (PAG; 3.5 mm posterior and 0.2 mm lateral to the bregma). The electrode array was directly implanted into the brain tissue, with electrodes inserted at varying depths (0.8 mm into S1, 2.0 mm into ACC, 2.5 mm into PL, 2.8 mm into PAG, 3.0 mm into THL, and 4.4 mm into NAc and AMY). Two electromyogram (EMG) electrodes were implanted in the dorsal neck area. In addition, stainless steel screws were positioned on the skull above the olfactory bulb and cerebellum, serving as recording electrodes for respiratory signals and ground/reference electrodes, respectively. All wires and electrode arrays were securely attached to the skull using dental cement. After completing all surgical procedures, anesthesia was discontinued, allowing the animals to awaken naturally. After surgery, each mouse was housed with free access to water and food while undergoing daily observations.

#### 2.1.3. Electrophysiological Recording and Histological Analysis to Confirm Electrode Placement

The mice were connected to the recording equipment via a Cereplex M (Blackrock Microsystems, Salt Lake City, UT, USA), a digitally programmable amplifier for electrophysiological signal recording. The head stage output was then transmitted to the Cereplex Direct recording system, a data acquisition system, using a lightweight multiwire tether and a commutator.

The mice were euthanized with an overdose of urethane/α-chloralose, followed by intracardial perfusion with 4% paraformaldehyde in phosphate-buffered saline (pH 7.4) and subsequent decapitation. After dissection, the brains were fixed overnight in 4% PFA and equilibrated with 20% and 30% sucrose in phosphate-buffered saline each overnight. Frozen coronal sections (50 µm) were obtained using a microtome, and the resulting serial sections were mounted and subjected to cresyl violet staining. For cresyl violet staining, the slices were water-rinsed, stained with cresyl violet, and overslipped using a hydrophobic mounting medium (Marinol). The positions of all electrodes were verified by identifying the corresponding electrode tracks in the histological tissue using an optical microscope (All-in-One Fluorescence Microscope BZ-X810, Keyence, Itasca, IL, USA).

#### 2.1.4. Simultaneous LFP Recording of Seven Brain Regions during Quiescent Periods

**Figure 1:**
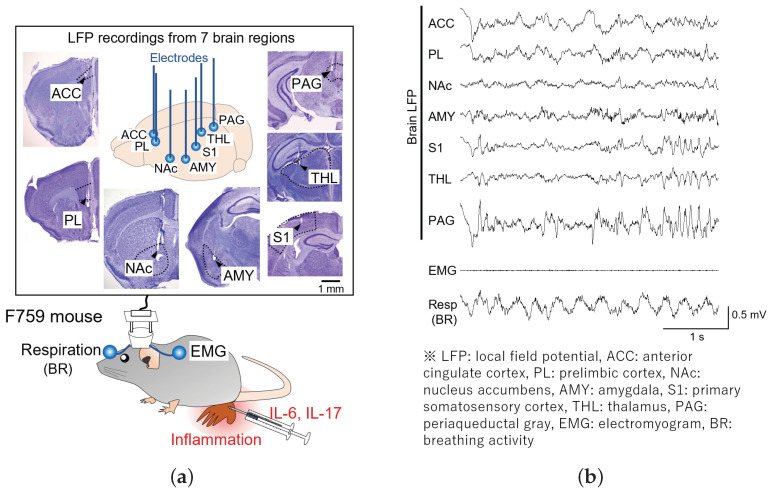
Simultaneous LFP recordings from seven brain regions of a freely moving mouse. At the bottom of (**a**), the electrophysiological signals, including brain LFP signals, an EMG signal, and a respiratory signal (BR), were obtained from an F759 mouse. At the top of (**a**), the histological confirmation of recording sites in ACC, PL, NAc, AMY, S1, THL, and PAG can be seen, as observed in the cresyl-stained sections. The dotted lines outline the contours of the brain regions, and the arrowheads indicate electrode tracks. (**b**) Representative LFP signals from ACC, PL, NAc, AMY, S1, THL, and PAG and EMG and respiratory signals. Quiescent periods were manually identified on the basis of nearly silent EMG signals.

LFP signals were simultaneously recorded for over 5 h from various brain regions, including ACC, PL, NAc, AMY, S1, THL, and PAG, in freely moving mice (Figure 1a). EMG signals were recorded using electrodes implanted in the dorsal neck area to assess the movements of the animals. Respiratory signals indicating breathing activity (BR) were recorded from the skull above the olfactory bulb. For analysis, LFP signals spanning 3600 s were manually extracted from quiescent periods identified when EMG signals exhibited minimal fluctuations (Figure 1b).

### 2.2. Feature Extraction

**Figure 2:**
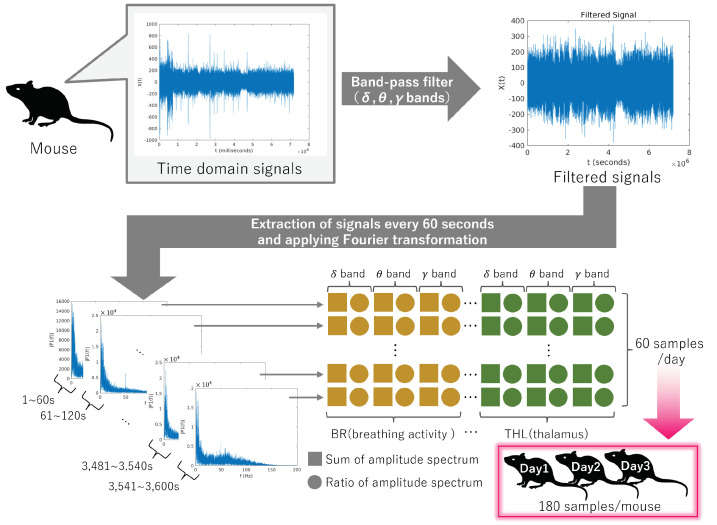
Overview of feature extraction from mice. Data acquisition spanned 1 h per day for three consecutive days. The 1 h data were divided into 1 min intervals, yielding 180 samples from each mouse.

These electrical signals were sampled at 2 kHz. In previous studies [27,28], the effectiveness of signals within the delta (1–4 Hz), theta (6–10 Hz), and gamma (40–100 Hz) bands was demonstrated. Corresponding band-pass filters were applied to the original signals to extract these bands. This experiment recorded data from a resting-state mouse over a 1 h period. A Hamming window was applied to segment the data into 1 min intervals. Fourier transformation was then applied to the segmented data to calculate each band’s amplitude spectrum and ratio to the entire signal. Consequently, 60 samples of six-dimensional signals (=3 bands × 2 types of data) were obtained for each mouse. Furthermore, signals were collected from mice three times at 3-day intervals, resulting in 180 samples per mouse. An overview of the feature calculation is shown in Figure 2.

## 3. Approach for Presymptomatic Disease Detection of Rheumatoid Arthritis

In Section 3.1, we detail the process of complementing missing brain activity information. In Section 3.2, we apply sMVCCA to the complemented brain activity information and label the information so as to indicate whether the mice are F759 or wild-type. Subsequently, we identify brain regions highly associated with presymptomatic mice. Finally, we confirm the high accuracy of presymptomatic detection of RA by classifying two types of mice using brain activity information obtained from regions strongly linked to presymptomatic mice in Section 3.3.

### 3.1. Completion of Missing Data

**Figure 3:**
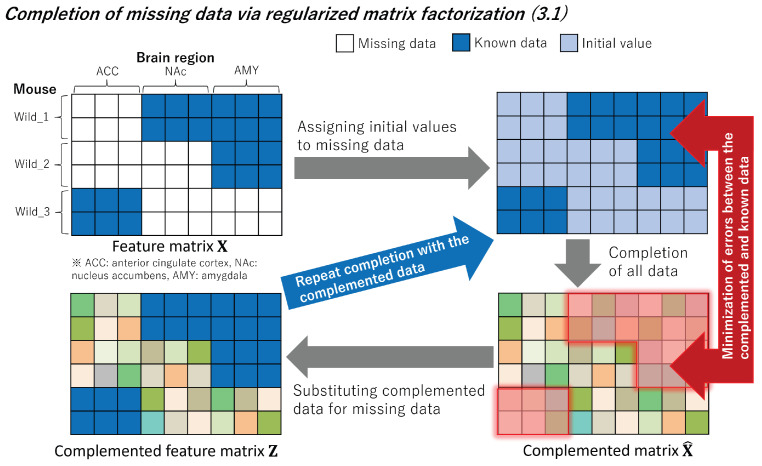
We initialize the missing data in the feature matrix X with initial values. We aim to minimize errors using known data by applying regularized matrix factorization to the obtained matrix. Subsequently, the missing data are replaced with complemented data, and this process is repeated.

In order to complement the missing data, we employed regularized matrix factorization. Figure 3 provides an overview of the complementation process. By considering the brain features $x_{n,m}\in R^{D}$ of the *m*-th brain region (m=1,2,…M;M being the number of brain regions) of the *n*-th mouse (n=1,2,…,N;N being the number of mice), we define the feature matrix $X\in R^{N\times (D\times M)}$ as follows:

(1) $$\begin{array}{c} X=\left[\begin{array}{cccc} x_{1,1}^{⊤} & x_{1,2}^{⊤} & \cdots & x_{1,M}^{⊤}\\ x_{2,1}^{⊤} & x_{2,2}^{⊤} & \cdots & x_{2,M}^{⊤}\\ \vdots & \ddots & \; & \vdots \\ x_{N,1}^{⊤} & x_{N,2}^{⊤} & \cdots & x_{N,M}^{⊤} \end{array}\right], \end{array}$$

where *D* represents the dimension of features derived from brain activity information and is set to 6, as explained in Section 2.2.

In order to complement the missing values in the feature matrix X, our method uses matrix factorization, which is a baseline for missing value complementation. In general matrix factorization, it is assumed that the feature matrix X containing missing values can be expressed as the product of the two matrices, P and Q, with *K*-dimensional latent features, as outlined below:

(2) $$\begin{array}{cc} X & \approx PQ^{T}\\ \; & =\hat{X}, \end{array}$$

where each row of P represents the strength of association between the mouse and the latent feature. In contrast, each row of Q represents the strength of association between the brain region feature and the latent feature. The (i,j)-th element of $\hat{X}$ is calculated using the feature vectors $p_{i}$ and $q_{j}$ from the matrices P and Q. By using this approach, we specifically compensate for missing values through matrix factorization as follows:

(3) $$\begin{array}{cc} {\hat{x}}_{ij} & =p_{i}^{T}q_{j}\\ \; & =\sum_{k=1}^{K}p_{ik}q_{kj}. \end{array}$$

Moreover, to determine the optimal P and Q, we minimize the squared error between the observed matrix X and the complemented matrix $\hat{X}$ as follows:

(4) $$\begin{array}{c} \arg\min_{P,Q}\sum_{i,j}{\left(x_{ij}-{\hat{x}}_{ij}\right)}^{2}=\arg\min_{P,Q}\sum_{i,j}{\left(x_{ij}-\sum_{k=1}^{K}p_{ik}q_{kj}\right)}^{2}. \end{array}$$

The loss of information about brain activity stems from malfunctions in the acquisition equipment, leading to the complete absence of specific brain regions. Because this loss is very different from the typically assumed random loss, it is difficult for general matrix factorization to compensate for it accurately. Our method introduces a regularized matrix factorization that incorporates bias terms for each mouse and brain region feature, accounting for the bias associated with missing values. In regularized matrix factorization, the optimal P and Q are estimated by solving the following equations:

(5) $$\begin{array}{c} \arg\min_{P,Q}\sum_{i,j}{\left(x_{ij}-{\hat{x}}_{ij}\right)}^{2}+\frac{\lambda }{2}\left({∥b_{\mathrm{mouse}}∥}^{2}+{∥b_{\mathrm{brain}}∥}^{2}+{∥p_{i}∥}^{2}+{∥q_{j}∥}^{2}\right), \end{array}$$

where $b_{\mathrm{mouse}}$ and $b_{\mathrm{brain}}$ represent the bias terms for each mouse and brain feature, respectively. Equation (5) can be solved using the gradient descent method, with each parameter updated as follows:

(6) $$\begin{array}{cc} b_{\mathrm{mouse}}^{\prime } & =b_{\mathrm{mouse}}+\alpha \left(2\mathrm{ave}\left(e_{i,*}\right)-\lambda b_{\mathrm{mouse}}\right), \end{array}$$

(7) $$\begin{array}{cc} b_{\mathrm{brain}}^{\prime } & =b_{\mathrm{brain}}+\alpha \left(2\mathrm{ave}\left(e_{*,j}\right)-\lambda b_{\mathrm{brain}}\right), \end{array}$$

(8) $$\begin{array}{cc} p_{ik}^{\prime } & =p_{ik}+\alpha \left(2e_{ij}q_{kj}-\lambda p_{ik}\right), \end{array}$$

(9) $$\begin{array}{cc} q_{kj}^{\prime } & =q_{kj}+\alpha \left(2e_{ij}p_{ik}-\lambda q_{kj}\right), \end{array}$$

(10) $$\begin{array}{cc} e_{ij} & =x_{ij}-\left(\mu +b_{\mathrm{mouse},i}+b_{\mathrm{brain},j}+p_{i}^{⊤}q_{j}\right). \end{array}$$

Finally, the (i,j)-th element of the complemented matrix $\hat{X}$ is predicted using the following equation:

(11) $$\begin{array}{c} {\hat{x}}_{ij}=\mu +b_{\mathrm{mouse},i}+b_{\mathrm{brain},j}+p_{i}^{⊤}q_{j}. \end{array}$$

Based on the aforementioned information, the missing elements in the feature matrix X are replaced with the corresponding elements in the predicted complemented matrix $\hat{X}$ to generate the complemented feature matrix Z, defined as follows:

(12) $$\begin{array}{cc} Z & =\left[\begin{array}{cccc} z_{1,1}^{⊤} & z_{1,2}^{⊤} & \cdots & z_{1,M}^{⊤}\\ z_{2,1}^{⊤} & z_{2,2}^{⊤} & \cdots & z_{2,M}^{⊤}\\ \vdots & \ddots & \; & \vdots \\ z_{N,1}^{⊤} & z_{N,2}^{⊤} & \cdots & z_{N,M}^{⊤} \end{array}\right] \end{array}$$

(13) $$\begin{array}{cc} \; & =[Z_{1},Z_{2},\cdots ,Z_{M}], \end{array}$$

where

(14) $$\begin{array}{c} z_{n,m}=\left\{\begin{array}{cc} {\hat{x}}_{n,m} & \left(\mathrm{if}\;m\text{-}\mathrm{th}\;\mathrm{brain}\;\mathrm{region}\;\mathrm{of}\;n\text{-}\mathrm{th}\;\mathrm{mouse}\;\mathrm{is}\;\mathrm{missing}\right)\\ x_{n,m} & \left(\mathrm{otherwise}\right) \end{array}\right.. \end{array}$$

Notably, $Z_{m}\in R^{N\times D}$ represents the complemented feature matrix obtained from the *m*-th brain region.

Furthermore, $b_{\mathrm{mouse}}$ and $b_{\mathrm{brain}}$ in Equation (5) are effective in complementing the missing values in specific brain regions because they introduce bias for each mouse and brain region. Consequently, applying regularized matrix factorization to the feature matrix enables accurate complementation of missing values in brain activity information.

### 3.2. Estimation of Important Brain Region

**Figure 4:**
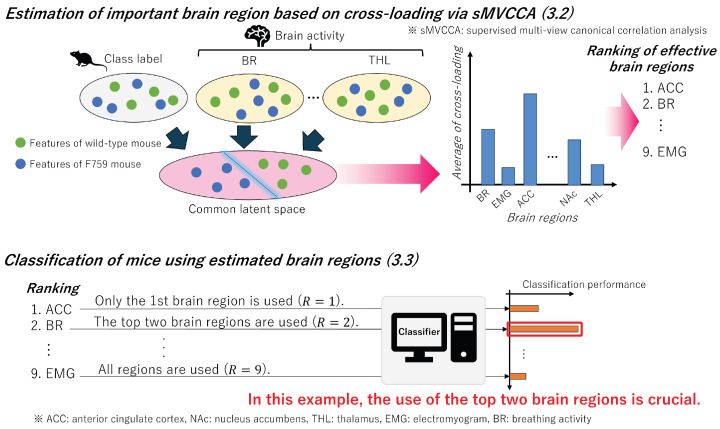
Overview of the process for identifying brain regions from complemented features that contribute to the classification of mice. In Section 3.2, a common latent space is constructed using sMVCCA, enabling the analysis of correlations between features from multiple brain regions and class labels. Because the features in this space are designed to be highly correlated with the class labels, the brain regions associated with the class labels are estimated by calculating the cross-loadings using these features. In addition, in Section 3.3, we further identify important brain regions by constructing various classification models using features ordered by cross-loading, starting with brain regions with the highest values.

In this subsection, we examine the potential correlation between class labels representing mouse types (i.e., F759 or wild-type) and feature vectors obtained from each brain region in the complemented feature matrix Z. Because multiple brain regions require comparison with class labels, we construct a latent variable model capable of handling multiview and supervised data. By using sMVCCA [18], which is designed to handle various types of information, we calculate cross-loadings to estimate important brain regions. The overall process is outlined at the top of Figure 4.

We estimate the projection vectors $v_{m^{\prime }}(m^{\prime }=\left\{1,2,\cdots ,M,l\right\};\;l$ begin the class label) by maximizing the following equation:

(15) $$\begin{array}{cc} \arg\max_{v_{m^{\prime }}}\sum_{m_{1}^{\prime }}\sum_{m_{2}^{\prime },\;m_{2}^{\prime }\neq m_{1}^{\prime }} & \frac{v_{m_{1}^{\prime }}^{⊤}C_{m_{1}^{\prime },m_{2}^{\prime }}v_{m_{2}^{\prime }}}{\sqrt{v_{m_{1}^{\prime }}^{⊤}C_{m_{1}^{\prime },m_{1}^{\prime }}v_{m_{1}^{\prime }}}\sqrt{v_{m_{2}^{\prime }}^{⊤}C_{m_{2}^{\prime },m_{2}^{\prime }}v_{m_{2}^{\prime }}}},\\ s.t.\;v_{m_{1}^{\prime }}^{⊤}C_{m_{1}^{\prime },m_{1}^{\prime }}v_{m_{1}^{\prime }}=1,\;\;\; & v_{m_{2}^{\prime }}^{⊤}C_{m_{2}^{\prime },m_{2}^{\prime }}v_{m_{2}^{\prime }}=1, \end{array}$$

where $C_{m_{1}^{\prime },m_{2}^{\prime }}=Z_{m_{1}^{\prime }}^{⊤}Z_{m_{2}^{\prime }}$ and $Z_{l}\in R^{N\times D_{l}}$, where $D_{l}$ is the number of class labels. In this study, because we used F759 and wild-type mice, we set $D_{l}$ to 2. Because the solution to the aforementioned problem is independent of the scale of $v_{m_{1}^{\prime }}$ and $v_{m_{2}^{\prime }}$, Equation (15) can be rewritten as follows:

(16) $$\begin{array}{cc} \arg\max_{v_{m^{\prime }}} & \sum_{m_{1}^{\prime }}\sum_{m_{2}^{\prime },m_{2}^{\prime }\neq m_{1}^{\prime }}v_{m_{1}^{\prime }}^{⊤}C_{m_{1}^{\prime },m_{2}^{\prime }}v_{m_{2}^{\prime }}. \end{array}$$

In our method, we define $V={\left[V_{1}^{⊤},V_{2}^{⊤},\cdots ,V_{M}^{⊤},V_{l}^{⊤}\right]}^{⊤}\in R^{\left(M\times D+D_{l}\right)\times D_{p}}$. Notably, $D_{p}\;(\leq$$\min(D,D_{l}))$ represents the dimension of the latent features obtained via sMVCCA. Equation (16) can be rewritten as follows:

(17) $$\begin{array}{cc} \arg\max_{V}\; & \mathrm{trace}\left(V^{⊤}\bar{C}V\right)\;s.t.\;V^{⊤}\underline{C}V=I, \end{array}$$

where

(18) $$\begin{array}{c} \bar{C}=\left[\begin{array}{ccccc} 0 & C_{1,2} & \cdots & C_{1,M} & C_{1,l}\\ C_{2,1} & 0 & \cdots & C_{2,M} & C_{2,l}\\ \vdots & \vdots & \ddots & \vdots & \vdots \\ C_{M,1} & C_{M,2} & \cdots & 0 & C_{M,l}\\ C_{l,1} & C_{l,2} & \cdots & C_{l,M} & 0 \end{array}\right], \end{array}$$

(19) $$\begin{array}{c} \underline{C}=\left[\begin{array}{ccccc} C_{1,1} & 0 & \cdots & 0 & 0\\ 0 & C_{2,2} & \cdots & 0 & 0\\ \vdots & \vdots & \ddots & \vdots & \vdots \\ 0 & 0 & \cdots & C_{M,M} & 0\\ 0 & 0 & \cdots & 0 & C_{l,l} \end{array}\right]. \end{array}$$

Finally, we solve the following generalized eigenvalue problem:

(20) $$\begin{array}{c} \bar{C}V=\epsilon \left(\underline{C}+\eta I\right)V, \end{array}$$

where ϵ denotes an eigenvalue, and η denotes a regularization parameter. The optimal projection ${\hat{V}}_{m^{\prime }}$ for the feature transformation is obtained by solving the aforementioned problem. The matrix ${\hat{V}}_{m^{\prime }}$ is constructed using the eigenvectors of the $D_{p}$-largest eigenvalues. We can then calculate the projected features as follows:

(21) $$\begin{array}{c} {\hat{Z}}_{m^{\prime }}=Z_{m^{\prime }}{\hat{V}}_{m^{\prime }}\in R^{N\times D_{p}}, \end{array}$$

where ${\hat{Z}}_{m^{\prime }}={\left[{\hat{z}}_{m^{\prime }}^{1},{\hat{z}}_{m^{\prime }}^{2},...,{\hat{z}}_{m^{\prime }}^{N}\right]}^{⊤}$. Consequently, by concatenating these projected features for each brain region, we can obtain the projected features ${\hat{z}}^{n}={\left[{\left({\hat{z}}_{1}^{n}\right)}^{⊤},{\left({\hat{z}}_{2}^{n}\right)}^{⊤},\cdots ,{\left({\hat{z}}_{M}^{n}\right)}^{⊤}\right]}^{⊤}$ of the *n*-th sample and construct the classifiers in Section 3.3.

In addition to estimating projected features, we calculated cross-loadings to identify brain regions important for classifying F759 and wild-type mice using sMVCCA. The cross-loading between the two features, g and h, can be calculated using Pearson’s correlation coefficient as follows:

(22) $$\begin{array}{c} Corr\left(g,h\right)=\frac{\sum_{i=1}^{N}\left(g_{i}-\bar{g}\right)\left(h_{i}-\bar{h}\right)}{\sqrt{\sum_{i=1}^{N}{\left(g_{i}-\bar{g}\right)}^{2}}\sqrt{\sum_{i=1}^{N}{\left(h_{i}-\bar{h}\right)}^{2}}}, \end{array}$$

where g and h represent the features, and $\bar{g}$ represents the average of g. In order to calculate the cross-loading between the *m*-th brain region and the class label, we set g to $z_{m,d}\in R^{N}\;\left(d=1,2,\cdots ,D\right)$. Notably, the *m*-th brain features are $z_{m}={\left[z_{m,1}^{⊤},z_{m,2}^{⊤},\cdots ,z_{m,D}^{⊤}\right]}^{⊤}$. In addition, h is set to the projected label feature ${\hat{z}}_{l}^{1}\in R^{N}$, where ${\hat{z}}_{l}^{1}$ is the feature with the highest eigenvalue obtained via sMVCCA. Consequently, the cross-loading $CL_{m}$ of the *m*-th brain region is computed by averaging the cross-loadings calculated from each dimension, *d*, and the projected label feature as follows:

(23) $$\begin{array}{c} CL_{m}=\sum_{d=1}^{D}Corr\left(z_{m,d},{\hat{z}}_{l}^{1}\right). \end{array}$$

If $CL_{m}$ is high, the *m*-th brain region will likely effectively classify mice.

The aforementioned process allows us to calculate the cross-loading between the class labels and features from each brain region in the common latent space. Therefore, effective brain regions can be estimated by computing cross-loading, $CL_{m}$.

### 3.3. Classification of Mice

In this section, by using the cross-loading calculated in Section 3.2, we arrange brain regions in descending order of their values and construct classifiers using the selected features. By using the constructed classifiers, we compared the classification performance of mice to identify an effective combination of brain regions. Essentially, this process allows us to identify those brain regions crucial for the presymptomatic detection of RA. The outlined flow is shown at the bottom of Figure 4.

In order to construct the classifier, we arrange the *M* brain regions in descending order on the basis of their cross-loading values and select up to the *R*-th highest cross-loading brain region. We then redefine the features ${\hat{z}}^{n}\in R^{D_{p}\cdot R}$ of the *n*-th sample as follows:

(24) $$\begin{array}{c} {\hat{z}}^{n}=\left[{\hat{z}}_{1}^{n^{⊤}},{\hat{z}}_{2}^{n^{⊤}},\cdots ,{\hat{z}}_{R}^{n^{⊤}}\right]. \end{array}$$

The analysis also assesses the robustness of the results by applying the obtained ${\hat{z}}^{n}$ to multiple classifiers. Specifically, four classifiers, namely, linear discriminant analysis (LDA), K-nearest neighbor (KNN), support vector machine (SVM) [29], and extreme learning machine (ELM) [30], are employed. The LDA model reduces within-class variance and increases between-class variance. The KNN model classifies target data on the basis of the Euclidean distance between features. The SVM model maximizes the distance, termed the margin, between the features of each class from the discriminative boundary distinguishing the classes. These three models are baseline and traditional classification models, which are commonly used in studies involving brain activity information, where acquiring a large amount of data is challenging. As the fourth classifier in this analysis, we opted for a neural network-based model. On the basis of a report that a simple multilayer perceptron can be effective, even with a small training dataset, we used an ELM model with one hidden layer. The classification performance of features, arranged according to their cross-loading values, is comparable, elucidating the combination of brain regions that are effective for detecting presymptomatic mice.

## 4. Experimental Conditions

**Figure 5:**
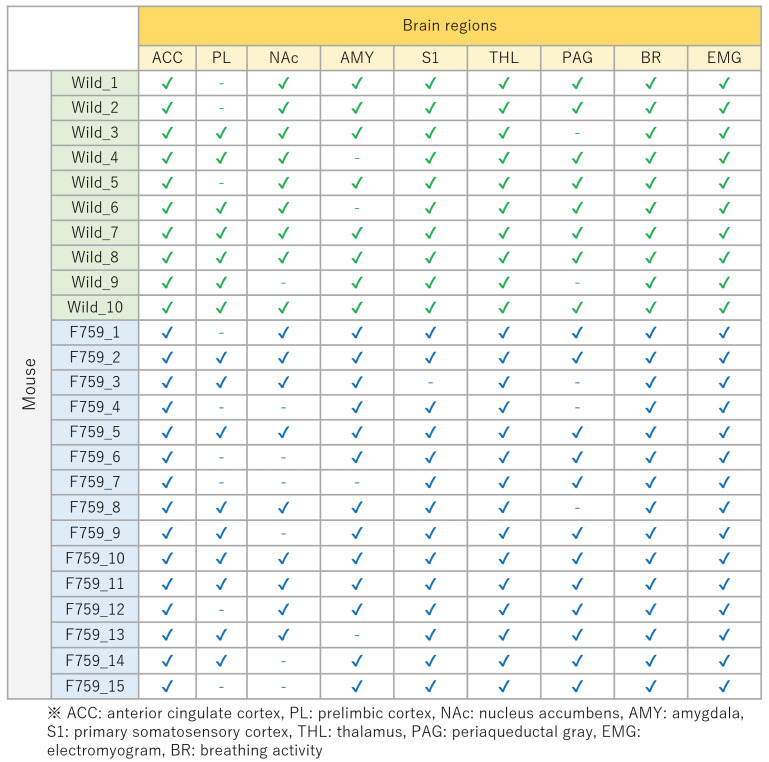
Relationship between each mouse and the missing brain region. “✓” indicates acquired data, and “-” indicates missing data.

In this experiment, we used 10 wild-type and 15 F759 mice. We attempted to obtain data from nine brain regions (i.e., M=9), some of which are missing. The relationship between each mouse and the missing data is shown in Figure 5. Figure 5 shows the relationship between the acquired data (“✓”) and the missing data (“-”). As shown in Figure 5, approximately 11.6% of the total data were missing. One way to address missing data is to exclude mice with missing data from the analysis. However, as shown in Figure 5, the method of excluding mice from the analysis is inappropriate because 18 out of 25 of the mice have missing data. Therefore, it was necessary to establish a method based on the assumption of missing data, and this study focused on data completion. These missing data were then complemented using the regularized matrix factorization introduced in Section 3.1.

The details of the parameters used in our method are as follows. The dimension, *D*, of the features obtained from each brain region was 6. α and λ, which were used in the regularized matrix factorization, were $2.0\times 10^{-5}$ and $1.0\times 10^{-5}$, respectively. η in sMVCCA was set to 0.01. The number of neighbors for the KNN model was set to 9, and a linear kernel was adopted as the kernel function for the SVM model. The ELM model is a three-layer neural network with 1000 nodes in the hidden layer.

For evaluation, we adopted the leave-mouse-out approach, where 24 out of 25 mice were used for training and 1 mouse was used for testing. The classifiers were constructed in the order of cross-loading, and the parameter *R*, which determines the number of features to be used, was varied from 1 to 9 in the experiments.

## 5. Results

**Figure 6:**
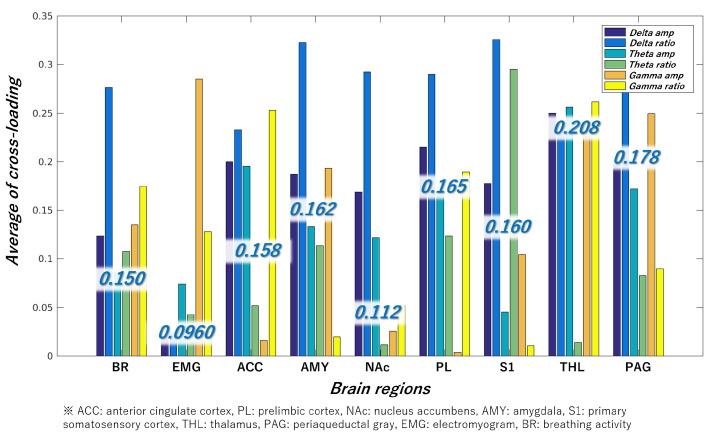
Cross-loading between data from nine brain regions and class labels. “Delta”, “theta”, and “gamma” indicate frequency bands. The terms “amp” and “ratio” indicate the amplitude spectrum and its ratio, respectively.

Figure 6 illustrates the cross-loading between the features obtained from the nine brain regions and class labels. A higher cross-loading indicates a greater likelihood that the brain region contributes to mouse classification. Because each brain region has six types of features, six cross-loadings are calculated from one brain region. Consequently, the average value of these cross-loadings is presented in blue letters in Figure 6 as the cross-loading of the target brain region. These results suggest that THL and PAG effectively detect presymptomatic disease in RA. The “Delta ratio” tended to be high even when the average cross-loading was low, suggesting that features in the 1–4-Hz frequency range were more important than those in other frequency ranges.

Subsequently, by comparing the magnitude of the cross-loading in Figure 6, it can be confirmed that the values of THL, PAG, PL, AMY, S1, ACC, BR, NAc, and EMG are larger in that order. Therefore, we performed mouse classification using these brain regions sequentially.

**Figure 7:**
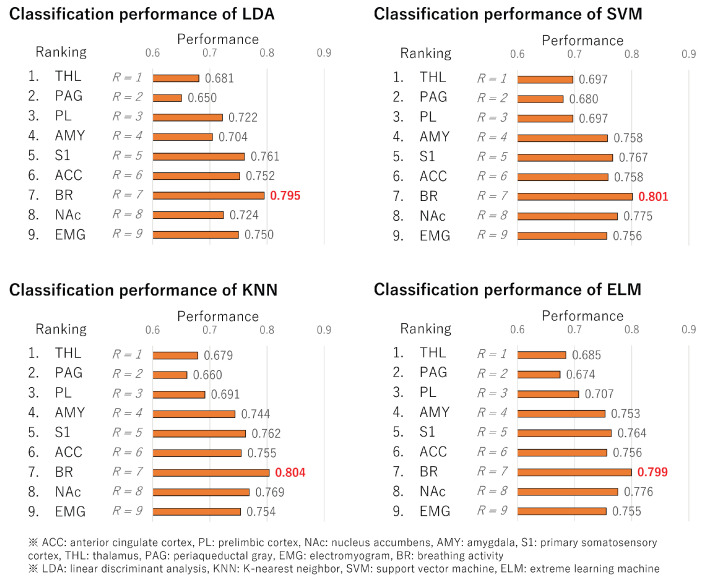
Classification performance of LDA, KNN, SVM, and ELM models. The results were obtained from classifiers constructed using the top *R*-ranked brain regions.

The LDA, KNN, SVM, and ELM classification results are presented in Figure 7. The term “Ranking” in Figure 7 represents the brain regions sorted in the order of cross-loading. A common finding from these results is that the highest accuracy is achieved when the top seven brain regions are used for all classifiers. In other words, brain regions other than EMG and NAc are effective.

Moreover, these findings indicate that classification performance improves when the top seven regions are included. In essence, there are correlations between cross-loadings and classification performance. Therefore, cross-loading is a highly effective method for estimating crucial brain regions without constructing classifiers.

## 6. Discussion

**Figure 8:**
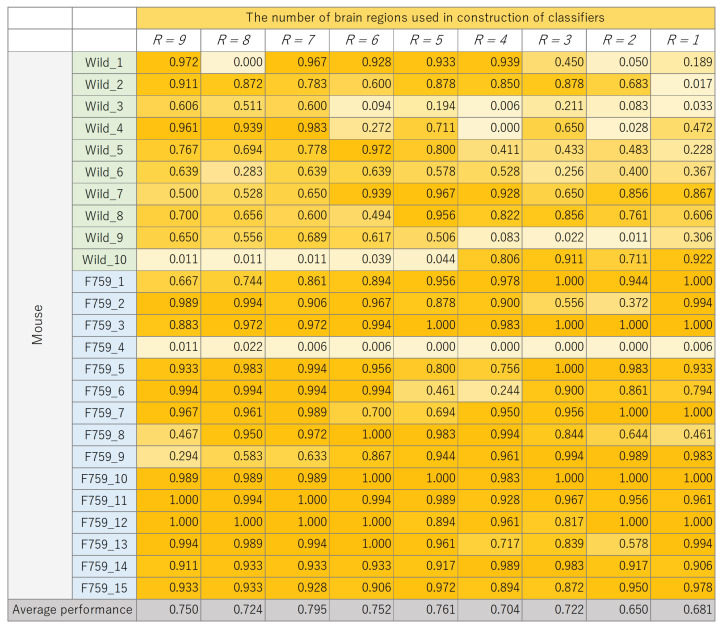
Classification performance of each mouse, with changes in the number of brain regions, *R*, used to construct linear discriminant analysis (LDA).

**Figure 9:**
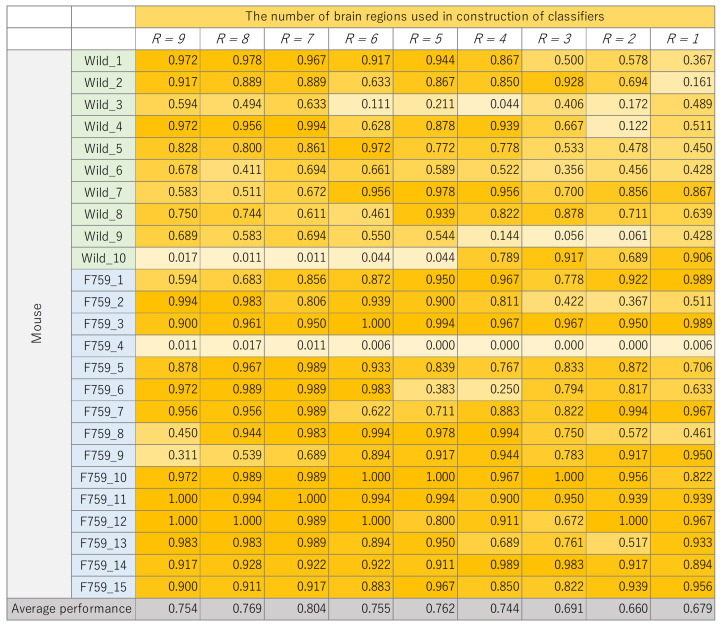
Classification performance of each mouse, with changes in the number of brain regions, *R*, used to construct K-nearest neighbor (KNN).

**Figure 10:**
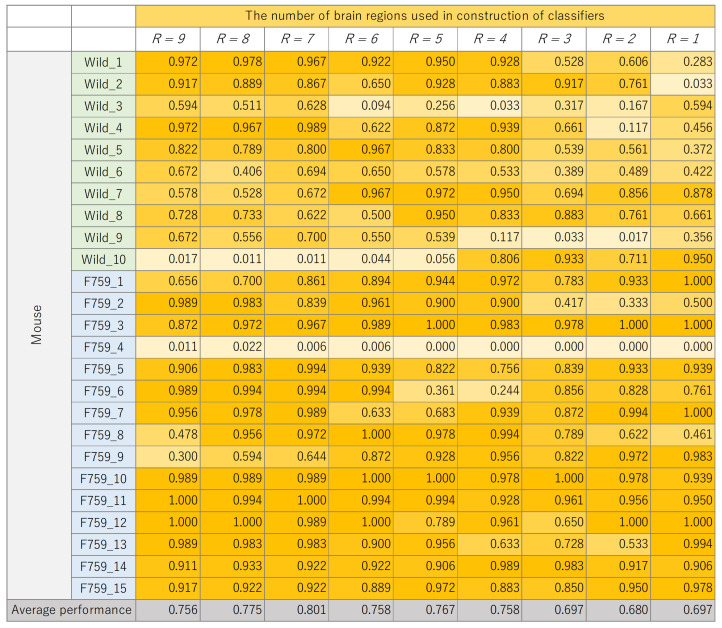
Classification performance of each mouse, with changes in the number of brain regions, *R*, used to construct support vector machine (SVM).

**Figure 11:**
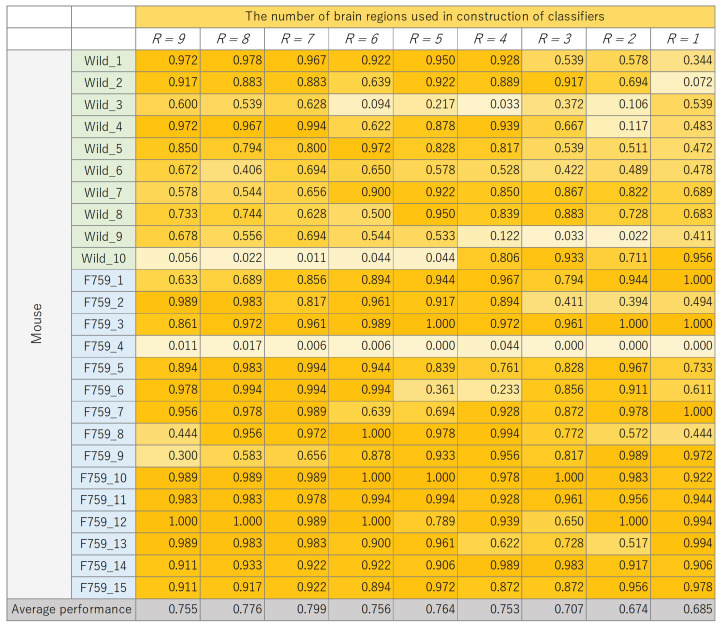
Classification performance of each mouse, with changes in the number of brain regions, *R*, used to construct extreme learning machine (ELM).

The classification results for each mouse, where the number of brain regions, *R*, was varied, are presented in Figure 8, Figure 9, Figure 10 and Figure 11. These figures show the mouse type on the vertical axis and the number of brain regions used to construct the classifier on the horizontal axis. The average performance at the bottom corresponds to the mean classification performance across all mice, which corresponds to the values presented in Figure 7. Figure 8, Figure 9, Figure 10 and Figure 11 provide a detailed breakdown of Figure 7. A generally consistent trend is observed in all figures. Specifically, the classification performance of wild-type mice tends to be lower than that of F759 mice. This discrepancy can be attributed to class imbalance, reflecting instability in learning due to the larger number of F759 mice than wild-type mice. Moreover, accuracy is notably low when R=1 and R=2, indicating the difficulty of achieving highly accurate classification, even when features from brain regions with high cross-loading are used. Conversely, as *R* increases, the overall accuracy improves, with some mice surpassing 90%.

However, even with an increase in *K*, no improvement in classification performance is seen for certain mice, such as wild_10 and F759_4. We attribute this phenomenon to inherent individual differences in biological information processing. It is conceivable that these differences pose challenges in accurately calculating potential correlations using sMVCCA. The acquired data will likely have a different distribution than the other data. In order to address this issue, potential strategies include excluding mice with different data distributions or removing noise on the basis of data characteristics. However, excluding specific specimens from experiments involving biological entities, such as mice, is not advisable because of the lack of data. Furthermore, elucidating data characteristics is a heuristic and impractical approach. Therefore, when constructing a common latent space, an effective solution is to compare the distribution of the 180 samples within a mouse with the distribution of samples across different mice and introduce a learning mechanism capable of bridging these distributional differences. By understanding differences in data characteristics, features useful for classification can be obtained, which is expected to further improve performance.

## 7. Conclusions

In this study, we analyzed brain activity using information obtained from mice to detect presymptomatic RA. The novelty of this method is that it attempts to identify brain regions crucial for presymptomatic RA detection by achieving high-accuracy classification between wild-type and F759 mice using a combination of multivariate analysis and machine learning. We introduced a matrix factorization-based approach for data completion to solve the problem of missing brain activity data. Furthermore, we applied sMVCCA to the complemented brain activity information and class labels, calculating cross-loadings between each brain region and class label to identify relevant brain regions. By constructing multiple classifiers using brain regions selected on the basis of cross-loadings, we successfully identified brain regions that were effective for detecting presymptomatic RA. Experiments involving 25 mice revealed the efficacy of seven brain regions, excluding NAc and EMG.

In order to verify the versatility of our method, it is desirable to conduct experiments using data from other diseases. Because the accumulation of amyloid-β secreted in the brain is related to the detection of Alzheimer’s disease [31,32], it is not necessary to focus on only the electrical signals of the brain, which are targeted in this study. Therefore, future research will involve investigating datasets for detecting presymptomatic diseases other than Alzheimer’s and conducting additional experiments using these datasets.

## 8. Ethical Approvals

All experiments were approved by the Committee on Animal Experiments at Tohoku University (approval number: 2022 PhA-004). All experiments were conducted following the NIH guidelines for the care and use of animals.

## Data Availability

Data cannot be released.
